# A lifespan pooled analysis of 832 cases: characterizing the lifespan profile of clinical presentations and comorbidities in congenital pulmonary airway malformation

**DOI:** 10.1186/s13023-026-04464-6

**Published:** 2026-07-01

**Authors:** Xiao Cheng, Chao Meng, Lirong Nie, Zhiwen Li, Jufen Liu

**Affiliations:** 1 Institute of Reproductive and Child Health/National Health Commission Key Laboratory of Reproductive Health, School of Public Health, Peking University, Beijing, China; 2 Department of Epidemiology and Biostatistics, School of Public Health, Peking University, Beijing, China; 3 Department of Maternal Health Care, Beijing Haidian Maternal and Child Health Hospital, Beijing, China; 4 State Key Laboratory of Female Fertility Promotion, Peking University, Beijing, China

**Keywords:** CPAM, Congenital lung malformation, Comorbidity, Clinical presentation, Age groups, Malignancy, Lifelong care

## Abstract

**Background:**

Congenital pulmonary airway malformation (CPAM) is a rare pulmonary developmental anomaly with heterogeneous clinical manifestations and associated comorbidities. While often diagnosed prenatally, its lifelong clinical spectrum and associated comorbidities remain poorly characterized, as existing evidence is fragmented across individual case reports and small series. To integrate and analyze individual patient data from published cases to delineate the age-stratified spectrum of comorbidities and clinical presentations of CPAM from fetal life to adulthood.

**Results:**

We performed a pooled analysis of individual case data extracted from 209 published articles during the period from 1992 to 2025. A total of 832 CPAM cases were stratified by age: fetus (*n* = 168), infant (*n* = 272), toddler (*n* = 70), child (*n* = 114), adolescent (*n* = 53), and adult (*n* = 155). In the 832 CPAM cases, a majority (66.6%; 95% CI 63.3%–69.8%) were asymptomatic at presentation, yet 37.4% (95% CI 34.1%–40.8%) had at least one documented comorbidity. Age-stratified analysis revealed significantly evolving profiles: malignant/pre-malignant lesions increased sharply with age (adults 23.9%; 95% CI 17.5%–31.3% vs. prenatal 2.9%; 95% CI 1.0%–6.9%, *p* < 0.001), while pulmonary sequestration was more frequent in fetal cases (9.5%). Asymptomatic presentation decreased from 89.4% prenatally to 48.4% in adulthood (*p* < 0.001), whereas infection-related symptoms, hemoptysis, and cough increased with age. Multivariate analysis identified symptomatic presentation at diagnosis as a strong, independent predictor of clinical outcome (aOR = 10.73, *p* = 0.004). Among the subset of 119 cases with genetic testing data, 63.0% (95% CI 53.7%–71.7%) harbored genetic findings of potential clinical significance.

**Conclusion:**

This large-scale pooled analysis demonstrates that CPAM exhibits a dynamic comorbidity and presentation spectrum across the lifespan, with increasing malignancy risk and symptomatic burden in adulthood. These findings challenge the perception of CPAM as solely a pediatric condition and argue for structured, lifelong follow-up. Specifically, we recommend: (1) age-stratified malignancy surveillance, with low-dose CT screening considered for adults with residual lesions; (2) transition from pediatric to adult care protocols that include patient education on symptom monitoring (e.g., new cough, hemoptysis); and (3) prioritized intervention and genetic counseling for symptomatic patients and those with identified genetic variants, given their higher risk of complications. Implementing these targeted strategies is crucial to optimize long-term outcomes.

**Supplementary Information:**

The online version contains supplementary material available at https://doi.org/10.1186/s13023-026-04464-6.

## Introduction

Congenital pulmonary airway malformation (CPAM), previously known as congenital cystic adenomatoid malformation, is a rare developmental anomaly of the lung parenchyma and airways, with an estimated incidence ranging from 1 in 10,000 to 1 in 35,000 live births [1–3]. It is characterized by abnormal proliferation of bronchiolar-like structures and cystic spaces, which may vary in size and distribution [4]. Although often detected prenatally during routine ultrasound examinations, CPAM can remain asymptomatic until later in life or present with a wide spectrum of respiratory and non-respiratory manifestations [5, 6]. This clinical heterogeneity is reflected in its histological spectrum, classified primarily by the Stocker system, which ranges from large cysts (Type I) to predominantly solid lesions (Type III) at the beginning, with distinct implications for management and prognosis [7]. With the improvement of examination techniques, the classification of CPAM has been expanded to five types, namely type 0 to type 4 [4].

Despite being one of the most common congenital lung lesions, CPAM remains a clinically heterogeneous and poorly understood entity, particularly in terms of its long-term natural history and associated comorbidities [8]. Historically, CPAM has been framed predominantly as a pediatric surgical disease, with clinical and research focus centered on prenatal diagnosis, postnatal management in infancy, and early childhood surgical outcomes [9–12]. However, a significant number of individuals with CPAM survive into adulthood [13–15], either diagnosed later in life or carrying a known childhood diagnosis [16]. This demographic shift raises critical, unanswered questions regarding the evolving risk of long-term complications, including recurrent infections [17], pneumothorax [18, 19], and notably, malignant transformation [20]—a concern underscored by reported associations with mucinous adenocarcinoma [21, 22], bronchioloalveolar carcinoma [23, 24], and other neoplasms [25, 26].

Current management guidelines, largely derived from pediatric experience, recommend surgical resection for symptomatic cases or lesions with concerning features but offer limited consensus on long-term surveillance for adolescents and adults, especially those managed conservatively [1, 15, 27, 28]. The critical lack of evidence is highlighted by the fact that the first large-scale prospective trial comparing management strategies in asymptomatic infants (the CONNECT trial) only began in 2023, with results not yet available [29]. Consequently, a fundamental knowledge gap persists: the systematic characterization of how comorbidity profiles and clinical presentations evolve from fetal life through adulthood is lacking. This gap directly hinders the development of evidence-based, age-stratified care pathways and creates uncertainty in counseling patients and families about lifelong prognosis and monitoring needs.

To address these pivotal evidence gaps, a comprehensive analysis that integrates data across the entire lifespan is urgently needed. Such a study must move beyond the traditional, fragmented view of isolated age groups (prenatal, pediatric, adult) and instead provide a holistic, longitudinal perspective on CPAM as a chronic condition with dynamic risks. By delineating the continuous evolution of comorbidities—with particular attention to the escalating malignancy risk—and clinical presentations, we can establish the empirical foundation necessary to inform tailored clinical approaches and surveillance protocols for each stage of life.

Therefore, we conducted a large-scale, retrospective pooled analysis of individual patient data from published cases spanning all age groups. The primary objectives of this study were to: (1) delineate the age-stratified spectrum of comorbidities and clinical presentations in CPAM from the prenatal period to adulthood; (2) identify key predictors of clinical outcome and (3) specifically analyze the association between CPAM and malignant transformation, including the exploration of underlying genetic findings, to inform risk stratification. By synthesizing data into a unified lifespan cohort, this study aims to challenge the prevailing pediatric-centric paradigm and provide the foundational evidence required to guide the development of standardized, lifelong management strategies for individuals with CPAM.

## Methods

### Study design and ethical considerations

This was a retrospective, pooled analysis designed to explore the spectrum of comorbidities and clinical presentations in patients with Congenital Pulmonary Airway Malformation (CPAM) by integrating individual patient data from published literature. To capture a comprehensive spectrum of CPAM presentations and outcomes across all ages, case data were assembled through a systematic literature review of published cases. This design allows for the integration of detailed, historically documented cases from a wide temporal and geographic range, including long-term outcomes and rare presentations that are critical for understanding the lifelong course of CPAM.

Ethical approval for this analysis of aggregated, anonymized literature data was not required as the study involved no direct patient contact or identifiable personal information. Informed consent was waived due to the retrospective nature of the study and the use of anonymized data.

### Data sources and case ascertainment

**Fig. 1:**
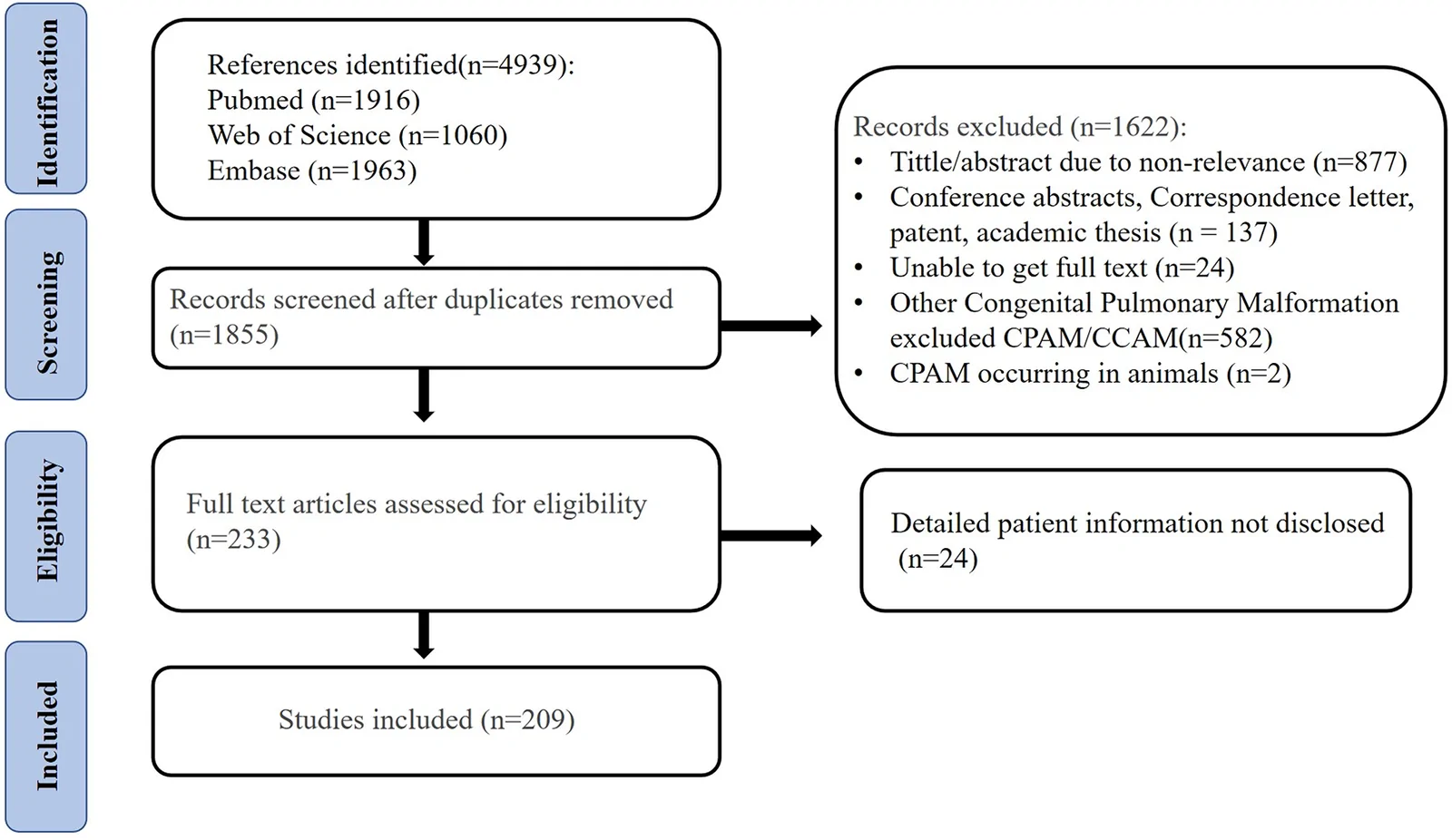
Study selection flowchart

Data were obtained from a pooled analysis of published individual patient data. A systematic search of the PubMed, Embase, and Web of Science databases was conducted for articles published from inception to December 2025, using keywords and MeSH terms related to CPAM (e.g., “congenital pulmonary airway malformation”, “congenital cystic adenomatoid malformation”, “CCAM”, “CCPM”). The initial search yielded 4,939 records. We included original articles, case series, cohort studies and review including cases information that reported individual patient-level data on demographics, diagnosis, presentation, comorbidities, or outcomes of CPAM. Correspondence letter, Conference abstract and studies without extractable case-level information were excluded. This process resulted in 209 articles from which individual patient data were meticulously extracted (Fig. 1). This pooled analysis approach, focusing on a systematic aggregation and synthesis of published individual case data, was chosen to construct a detailed lifespan cohort that captures the broadest possible disease spectrum—including asymptomatic, prenatal, and adult cases—as reported in the medical literature, thereby providing a foundational overview of CPAM’s clinical evolution.

To construct a comprehensive lifespan cohort that captures both the published disease spectrum and the often-underrepresented “real-world” population, data from both sources were integrated after a rigorous deduplication process. This approach allowed us to leverage the complementary strengths of each source: the literature-derived cohort provided detailed, historically documented cases including long-term outcomes and rare presentations, while the population-based registry captured unselected, contemporary cases—including asymptomatic and prenatally diagnosed individuals often missing from the literature—thus mitigating publication bias and providing a more generalizable sample. A unified dataset was then created by harmonizing variable definitions and removing duplicate records at the patient level where identifiers permitted.

### Case selection and inclusion criteria

From the pooled data, we included all cases that met the following criteria:

1. A confirmed diagnosis of CPAM based on established histopathological examination (post-resection or autopsy) or characteristic imaging findings (prenatal ultrasound, postnatal computed tomography) as documented in the source articles or registry records.
2. Availability of core data points, including age at primary diagnosis or presentation, and information on at least one of the following: clinical presentation, associated comorbidity, or basic demographic characteristic (sex, lesion location).
3. Cases were excluded if the diagnosis was ambiguous (e.g., unclear differentiation from other lung lesions like bronchopulmonary sequestration) or if essential data were completely lacking.

The final analytical cohort comprised 832 unique CPAM cases (including prenatal cases and postnatal cases).

### Data extraction and variable definitions

A standardized electronic form was used for data extraction. Variables collected included:

1. Demographics and Diagnosis: Age at primary diagnosis/ presentation, sex, laterality of the lesion (left, right, bilateral), and CPAM histological type (Stocker classification when available).
2. Temporal Context: Patients were categorized as “Prenatal CPAM” if the diagnosis was made before birth (primarily via fetal ultrasound), and “Postnatal CPAM” if diagnosed after birth. For detailed age-stratified analysis, cases were grouped into: Fetus (prenatal diagnosis), Infant (< 1 year), Toddler (1–2 years), Child (3–11 years), Adolescent (12–17 years), and Adult (≥ 18 years).
3. Clinical Presentation Spectrum: The primary documented mode of presentation was recorded and categorized (e.g., asymptomatic/incidental finding, respiratory distress, infection-related symptoms, cough, hemoptysis, chest pain, pneumothorax, prenatal complications).
4. Comorbidity Spectrum: All documented associated conditions were recorded and classified into major categories: malignant/pre-malignant lesions (e.g., mucinous adenocarcinoma, bronchioloalveolar carcinoma), pulmonary sequestration, cardiovascular anomalies, infections/inflammatory conditions, other cysts/structural anomalies, pneumothorax, and pleuropulmonary blastoma.
5. Management and Outcome: Primary treatment approach (surgical resection vs. conservative management) and documented outcome (alive/asymptomatic, died/termination/recurrence) were extracted where available.

### Statistical analysis

Data were analyzed using R software (version 4.4.0). Descriptive statistics were presented as frequencies and percentages for categorical variables. Group comparisons (e.g., Prenatal vs. Postnatal, across age groups) for categorical variables were performed using the Chi-square test or Fisher’s exact test, as appropriate. A two-sided p-value < 0.05 was considered statistically significant. To identify independent predictors of a composite adverse outcome (encompassing mortality, pregnancy termination for fetal anomaly, or disease recurrence), a multivariate logistic regression model was constructed, calculating adjusted odds ratios (aORs) with 95% confidence intervals (CIs). All analyses were conducted on the available data without imputation for missing values. Confidence interval for a binomial proportion were calculated using the Clopper–Pearson method.

## Results

### Cohort characteristics

The final cohort comprised 832 CPAM cases derived from a publication period from 1992 to 2025 and originated from multiple geographic regions, including Europe, Japan, South Korea, China, the United States, and, to a lesser extent, Latin America and Africa. The overall cohort exhibited a broad age distribution: 168 (20.2%) were diagnosed prenatally (Fetus), 272 (32.7%) in infancy (< 1 year), 70 (8.4%) in toddlerhood (1–2 years), 114 (13.7%) in childhood (3–11 years), 53 (6.4%) in adolescence (12–17 years), and 155 (18.6%) in adulthood (≥ 18 years). Baseline characteristics stratified by age group are detailed in Table 1.

**Table 1:** Baseline characteristics of patients with CPAM, stratified by age group

Characteristics	Age group of CPAM *n*(%)							
	Total (*n* = 832)	Fetus (*n* = 168)	Infant (*n* = 272)	Toddler (*n* = 70)	Child (*n* = 114)	Adolescent (*n* = 53)	Adult (*n* = 155)	*P* value
**Classification**								< 0.001*
0	1(0.1)	1(0.6)	0(0)	0(0)	0(0)	0(0)		
I	410(49.2)	62(36.9)	103(37.9)	40(57.1)	59(51.8)	35(66.0)	111(71.6)	
II	196(23.5)	17(10.1)	82(30.1)	18(25.8)	33(28.9)	12(22.6)	34(31.9)	
III	72(8.6)	35(20.8)	23(8.5)	4(5.7)	5(4.4)	1(1.9)	4(2.6)	
IV	11(1.3)	0(0.0)	5(1.8)	2(2.9)	0(0.0)	1(1.9)	3(1.9)	
Mixed	9(1.0)	1(0.6)	2(0.7)	1(1.4)	3(2.6)	1(1.9)	1(0.6)	
Undisclosed/Not available	133(15.9)	52(31.0)	57(21.0)	5(7.1)	14(12.3)	3(5.7)	2(1.4)	
**Sex**								< 0.001*
Female	311(37.3)	46(27.4)	99(36.4)	33(47.1)	57(50.0)	18(33.9)	64(41.2)	
Male	368(44.2)	40(23.8)	146(53.7)	36(51.4)	48(42.1)	35(66.1)	57(36.7)	
Undisclosed/Not available	153(18.3)	82(48.8)	27(9.9)	1(1.5)	9(7.9)	0(0)	34(21.9)	
**Location**								< 0.001*
Left	303(36.4)	59(35.1)	112(41.1)	12(17.1)	25(21.9)	20(37.7)	75(48.3)	
Right	327(39.3)	40(23.8)	126(46.3)	17(24.2)	37(32.4)	27(50.9)	66(42.5)	
Bilateral	22(2.6)	7(4.2)	7(2.5)	0(0)	3(2.6)	0(0.0)	5(3.2)	
Undisclosed/Not available	180(21.7)	48(28.6)	27(9.9)	41(58.7)	49(42.9)	6(11.4)	9(6.0)	
**Comorbidity**								< 0.001*
Mucinous adenocarcinoma	51(6.1)	5(1.6)	13(4.7)	3(4.2)	3(2.6)	10(18.8)	17(10.9)	
Bronchioloalveolar Carcinoma	34(4.1)	0(0)	4(1.4)	0(0)	8(7.0)	9(16.9)	13(8.3)	
Cardiac Disease	14(1.7)	2(1.2)	5(1.8)	0(0)	7(6.1)	0(0)	0(0)	
Other Pulmonary Comorbidity	46(5.5)	3(0.9)	11(4.0)	5(7.1)	9(7.8)	6(11.3)	12(7.7)	
Other Comorbidity	156(18.8)	50(29.8)	46(16.9)	6(8.5)	12(10.5)	9(16.9)	33(21.2)	
**Treatment**								< 0.001*
Surgical Treatment	597(71.7)	75(44.6)	242(89.0)	46(65.7)	58(50.8)	45(84.9)	131(84.5)	
Conservative Management	27(3.2)	10(5.9)	8(2.9)	2(2.8)	2(1.7)	0(0)	5(3.2)	
Undisclosed/Unknown	208(25.0)	83(49.5)	22(8.1)	22(31.5)	54(47.3)	8(15.1)	19(12.3)	
**Gene Test**								0.353
Mutation	119(14.4)	24(14.3)	55(20.2)	13(18.6)	8(7.0)	10(18.9)	9(5.8)	
No Mutation/Not available	713(85.6)	144(85.7)	217(79.8)	57(81.4)	106(93.0)	43(81.1)	146(94.2)	
**Outcome**								< 0.001*
Alive/Asymptomatic	523(62.8)	120(71.4)	154(56.6)	54(77.2)	82(71.9)	31(58.4)	82(52.9)	
Died/Termination/Recurrence	63(7.5)	37(22.1)	16(5.8)	2(2.8)	3(2.6)	0(0)	5(3.2)	
Undisclosed/Not available	246(29.7)	11(6.5)	102(37.6)	14(20.0)	29(25.5)	22(41.6)	68(43.9)	
**Presentation**								
Asymptomatic	555(66.6)	136(80.9)	170(62.5)	58(82.8)	90(78.9)	26(49.0)	75(48.3)	< 0.001*

*P value < 0.05

**Fig. 2:**
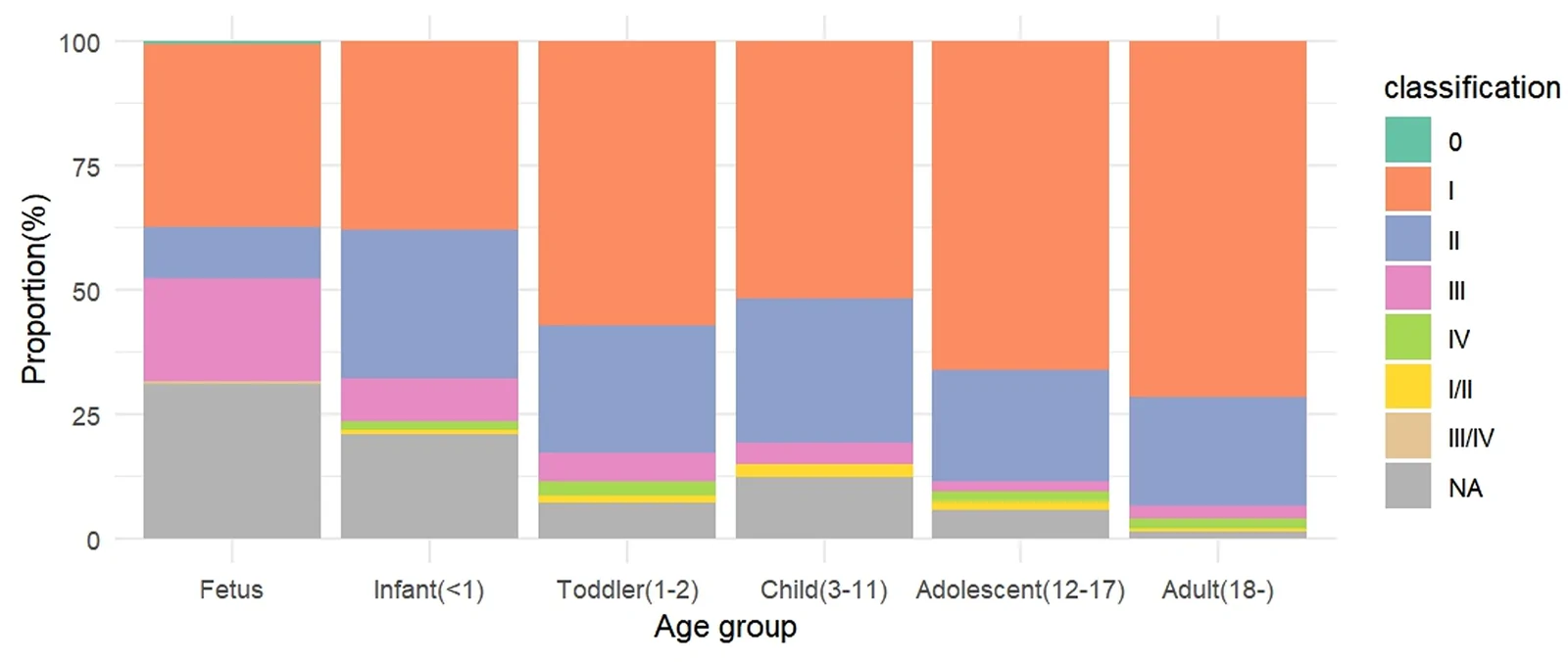
Distribution of CPAM classification by age group

Demographic and clinical profiles varied significantly across the lifespan (all *p* < 0.001). There was a male predominance (44.2%; 95% CI 40.8%–47.7%, female 37.3%, not available 18.3%) overall, which was most pronounced in adolescents (66.1%). Lesions were slightly more common on the right side (39.3%; 95% CI 36.0%–42.7%) than the left (36.4%; 95% CI 33.1%–39.8%), though this laterality pattern fluctuated with age. The distribution of CPAM histological subtypes (Stocker classification) showed a clear evolutionary pattern (Fig. 2). Type I lesions progressively increased in frequency from 36.9% (95% CI 29.5%–44.8%) in the fetal group to 71.6% (95% CI 63.9%–78.5%) in adults. In contrast, Type III lesions were most prevalent prenatally (20.8%) and became rare beyond infancy.

### Evolving spectrum of clinical presentation

The primary mode of presentation demonstrated profound age-dependent shifts (Table 2, Supplementary Table 1). The proportion of patients who were asymptomatic or diagnosed incidentally was highest prenatally (80.9%; 95% CI 74.3%–86.4%) and decreased significantly with age, falling to 48.4% (95% CI 40.2%–56.6%) in adults (*p* < 0.001).

**Table 2:** Spectrum of presentation with CPAM

Primary Presentation *n* (%)	Total CPAM cases (*n* = 832)	Prenatal CPAM (*n* = 168)	Postnatal CPAM (0–18) (*n* = 509)	Adult CPAM (> 18) (*n* = 155)	*P* value
**Respiratory Distress**	**54(6.5)**	**7(4.2)**	**46(9.0)**	**1(0.6)**	< 0.001*
**Asymptomatic / Incidental Finding**	**555(66.6)**	**136(80.9)**	**344(67.5)**	**75(48.4)**	< 0.001*
**Infection-Related Presentation**	**59(7.1)**	**4(2.4)**	**47(9.2)**	**8(5.2)**	< 0.001*
- Pneumonia / recurrent infection	54(6.5)	1(0.6)	45(8.8)	8(5.2)	
- Fever	5(0.6)	3(1.8)	2(0.4)	0(0)	
**Cough**	**36(4.3)**	**0(0)**	**23(4.5)**	**13(8.4)**	< 0.001*
- Cough alone / recurrent cough	21(2.5)	0(0)	13(2.6)	8(5.1)	
- Cough combined with other symptoms	15(1.8)	0(0)	10(2.0)	5(3.2)	
**Hemoptysis**	**17(2.1)**	**0(0.0)**	**7(1.4)**	**10(6.4)**	< 0.001*
- Hemoptysis alone	6(0.7)	0(0)	2(0.4)	4(2.6)	
- Hemoptysis with other symptoms	11(1.4)	0(0)	5(1.0)	6(3.8)	
**Prenatal / Fetal & Maternal Complications**	**21(2.5)**	**19(11.3)**	**2(0.4)**	**0(0)**	< 0.001*
- Fetal hydrops / History of fetal hydrops	8(0.9)	7(4.2)	1(0.2)	0(0)	
- Polyhydramnios / Oligohydramnios	4(0.5)	4(2.4)	0(0)	0(0)	
- Major structural / chromosomal anomalies	6(0.7)	6(3.6)	0(0)	0(0)	
- Maternal complications	3(0.4)	2(1.2)	1(0.2)	0(0)	
**Chest Pain / Discomfort**	**13(1.5)**	**0(0)**	**9(1.8)**	**4(2.6)**	0.006*
**Pneumothorax**	**10(1.2)**	**2(1.2)**	**7(1.4)**	**1(0.6)**	0.430
**Other Single Presentations**	**67(8.0)**	**0(0.0)**	**24(4.7)**	**43(27.7)**	< 0.001*

*P value < 0.05

Conversely, symptomatic presentations became more diverse and frequent postnatally. Infection-related presentations (e.g., pneumonia, recurrent infections) were reported in 9.2% of postnatal cases (7.1% overall, 95% CI 5.5%–9.1%) but were exceedingly rare prenatally (2.4%, 0.7%–5.4%, *p* < 0.001). Cough and hemoptysis were absent in the prenatal cohort but emerged as notable symptoms in postnatal life, with hemoptysis being particularly salient in adults (6.4%; 95% CI 3.1%–11.5% vs. 1.4% in 0–18 years, *p* < 0.001). Chest pain was exclusively a postnatal complaint (1.8% in 0–18 years; 2.6% in adults).

Prenatal presentations were predominantly related to fetal and maternal complications, such as fetal hydrops (4.2%) and polyhydramnios (2.4%), which were rare postnatally.

### Spectrum of comorbidities across the lifespan

**Fig. 3:**
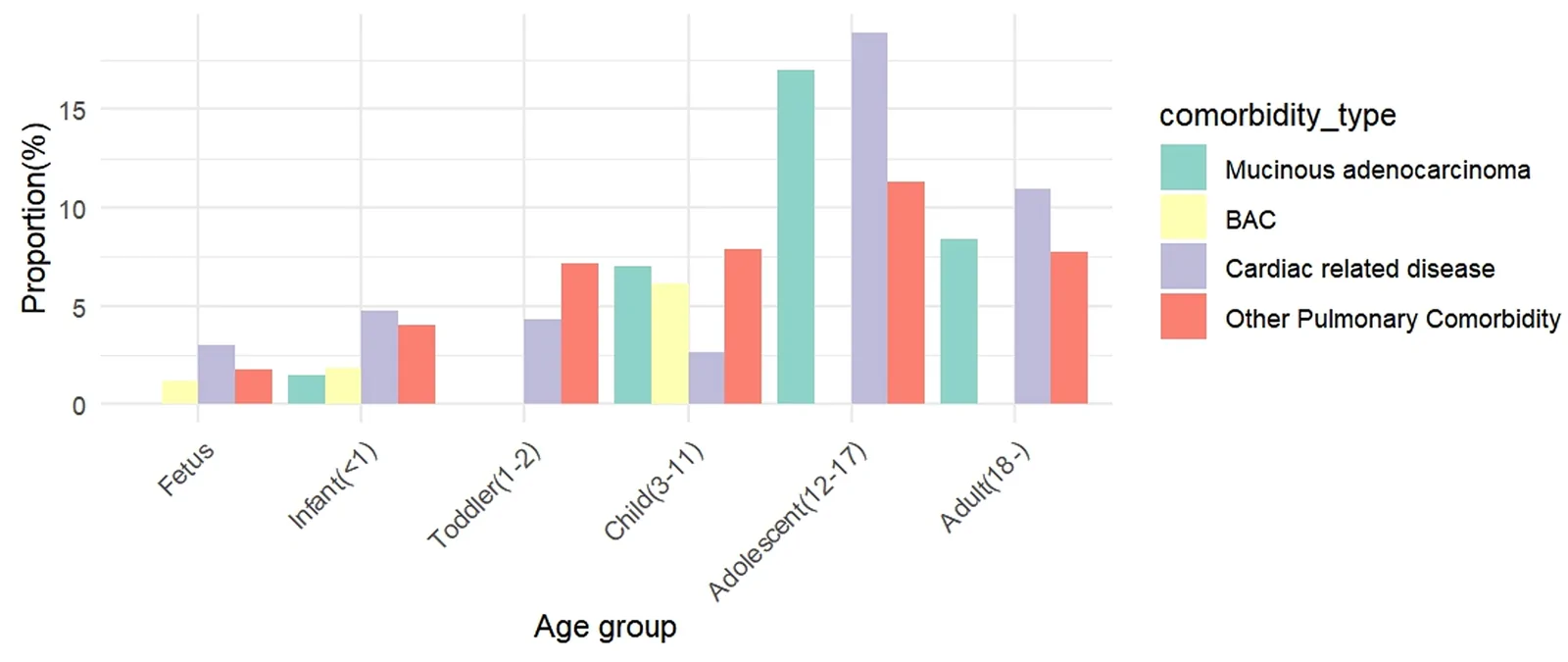
Distribution of cardiopulmonary comorbidities by age group

**Fig. 4:**
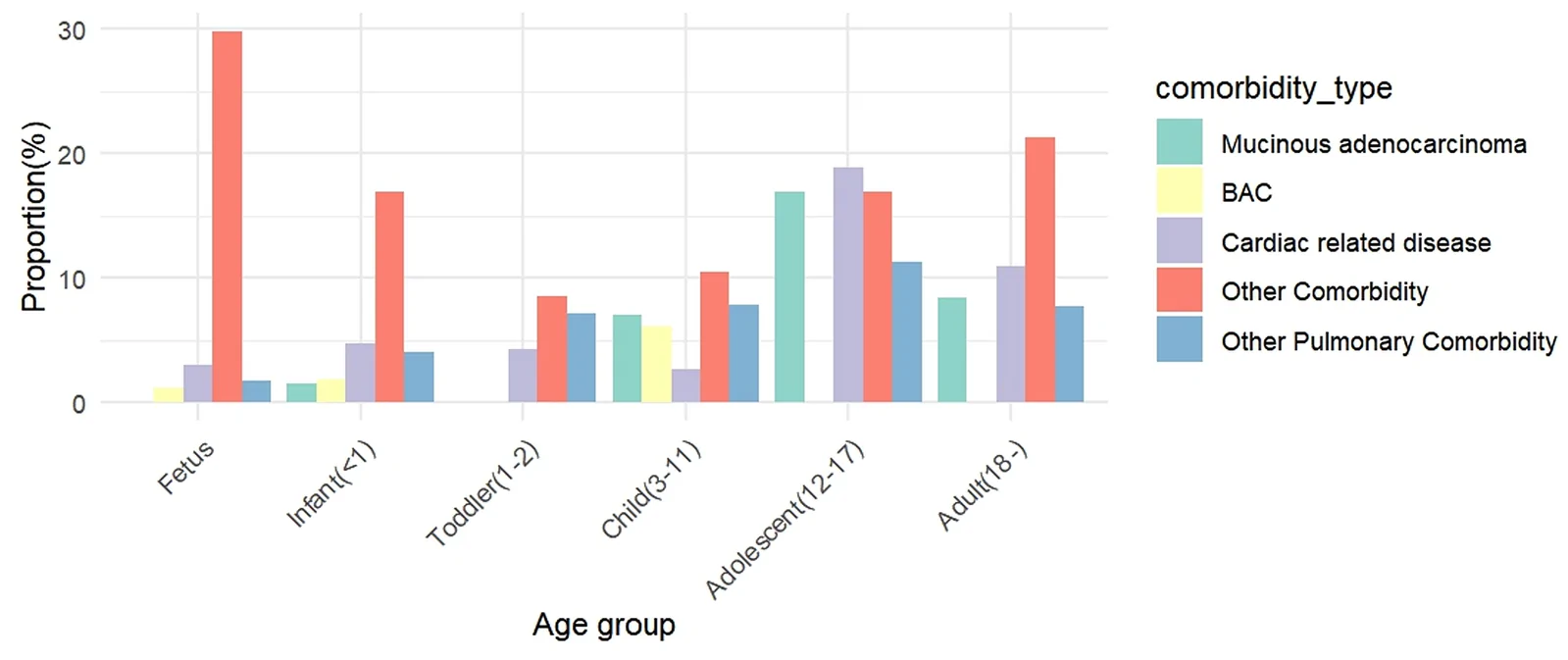
Distribution of comorbidities by age group

Comorbidity profiles evolved dramatically from the prenatal period through adulthood (Table 3, Supplementary Table 2, Figs. 3 and 4). The proportion of patients with no documented comorbidity was 58.3% (95% CI 50.4%–65.9%) prenatally but declined to 51.6% (95% CI 43.4%–59.7%) in adults (*p* < 0.001).

**Table 3:** Spectrum of comorbidities with CPAM

Comorbidity Event *n* (%)	Total CPAM cases (*n* = 832)	Prenatal CPAM (*n* = 168)	Postnatal CPAM (0–18) (*n* = 509)	Adult CPAM (> 18) (*n* = 155)	*P* value
**Malignant / Pre-malignant Lesions**	**81 (9.7)**	**5 (2.9)**	**39 (7.7)**	**37 (23.9)**	< 0.001*
**No Comorbidity**	**521(62.6)**	**98(58.3)**	**343(67.4)**	**80(51.6)**	< 0.001*
- Mucinous adenocarcinoma (MAC)	55 (6.6)	5 (2.9)	30 (5.9)	20 (12.9)	
- Bronchioloalveolar carcinoma (BAC)	26 (3.1)	0 (0.0)	13 (2.6)	13 (8.4)	
**Pulmonary Sequestration**	**34 (4.1)**	**16 (9.5)**	**16 (3.1)**	**2 (1.3)**	0.018*
- Extralobar pulmonary sequestration	16 (1.9)	10 (5.9)	6 (1.2)	0 (0.0)	
- Intralobar pulmonary sequestration	18 (2.2)	6 (3.6)	10 (2.0)	2 (1.3)	
**Cardiovascular Anomalies**	**19 (2.3)**	**2 (1.2)**	**16 (3.1)**	**1 (0.6)**	0.034*
- Congenital heart disease / malformation	11 (1.3)	2 (1.2)	9 (1.8)	0 (0.0)	
- Other cardiovascular anomalies	8 (1.0)	0 (0.0)	7 (1.4)	1 (0.6)	
**Infections & Inflammatory Conditions**	**25 (3.0)**	**0 (0.0)**	**13 (2.6)**	**12 (7.7)**	< 0.001*
- Pneumonia / lung abscess / empyema	10 (1.2)	0 (0.0)	7 (1.4)	3 (1.9)	
- Tuberculosis / Nontuberculous mycobacteria	5 (0.6)	0 (0.0)	1 (0.2)	4 (2.6)	
- Aspergillosis / Aspergilloma	5 (0.6)	0 (0.0)	2 (0.4)	3 (1.9)	
- Others (CMV, fungus colonization, etc.)	5 (0.6)	0 (0.0)	3 (0.6)	2 (1.3)	
**Other Cysts / Structural Anomalies**	**14 (1.6)**	**4 (2.4)**	**9 (1.8)**	**1 (0.6)**	0.320
- Bronchogenic / esophageal	7 (0.8)	3 (1.8)	4 (0.8)	0 (0.0)	
- Arachnoid / other cysts	7 (0.8)	1 (0.6)	5 (1.0)	1 (0.6)	
**Pneumothorax**	**11 (1.3)**	**0 (0.0)**	**9 (1.8)**	**2 (1.3)**	0.002*
- Spontaneous pneumothorax	6 (0.7)	0 (0.0)	5 (1.0)	1 (0.6)	
- Other / unspecified pneumothorax	5 (0.6)	0 (0.0)	4 (0.8)	1 (0.6)	
**Pleuropulmonary Blastoma (PPB)**	**5 (0.6)**	**0 (0.0)**	**5 (1.0)**	**0 (0.0)**	0.002*
**Asthma**	**5 (0.6)**	**0 (0.0)**	**2 (0.4)**	**3 (1.9)**	
**Other Single Comorbidities**	**137 (16.5)**	**43 (25.6)**	**76 (14.9)**	**17 (11.0)**	0.820

*P value < 0.05

Strikingly, malignant and pre-malignant lesions, including mucinous adenocarcinoma (MAC) and bronchioloalveolar carcinoma (BAC), exhibited a steep rise in prevalence with age. They were identified in only 2.9% (95% CI 1.0%–6.9%) of prenatal cases, 7.7% (95% CI 5.6%–10.3%) of pediatric (0–18 years) cases, and 23.9% (95% CI 17.5%–31.3%) of adult cases (*p* < 0.001). MAC was the most common malignancy across all groups.

Pulmonary sequestration, both extralobar and intralobar, showed an inverse pattern, being most frequent in the fetal group (9.5%) and becoming less common with increasing age (1.3% in adults, *p* = 0.018). Cardiovascular anomalies, primarily congenital heart disease, were most prevalent in childhood (6.1%) and were absent in the adult cohort (*p* = 0.034).

Infectious and inflammatory conditions (e.g., pneumonia, tuberculosis, aspergillosis) were absent prenatally but present in 2.6% of pediatric and 7.7% (95% CI 4.2%–12.8%) of adult cases (*p* < 0.001), highlighting a lifelong risk.

### Treatment patterns, outcomes, and predictive factors

**Fig. 5:**
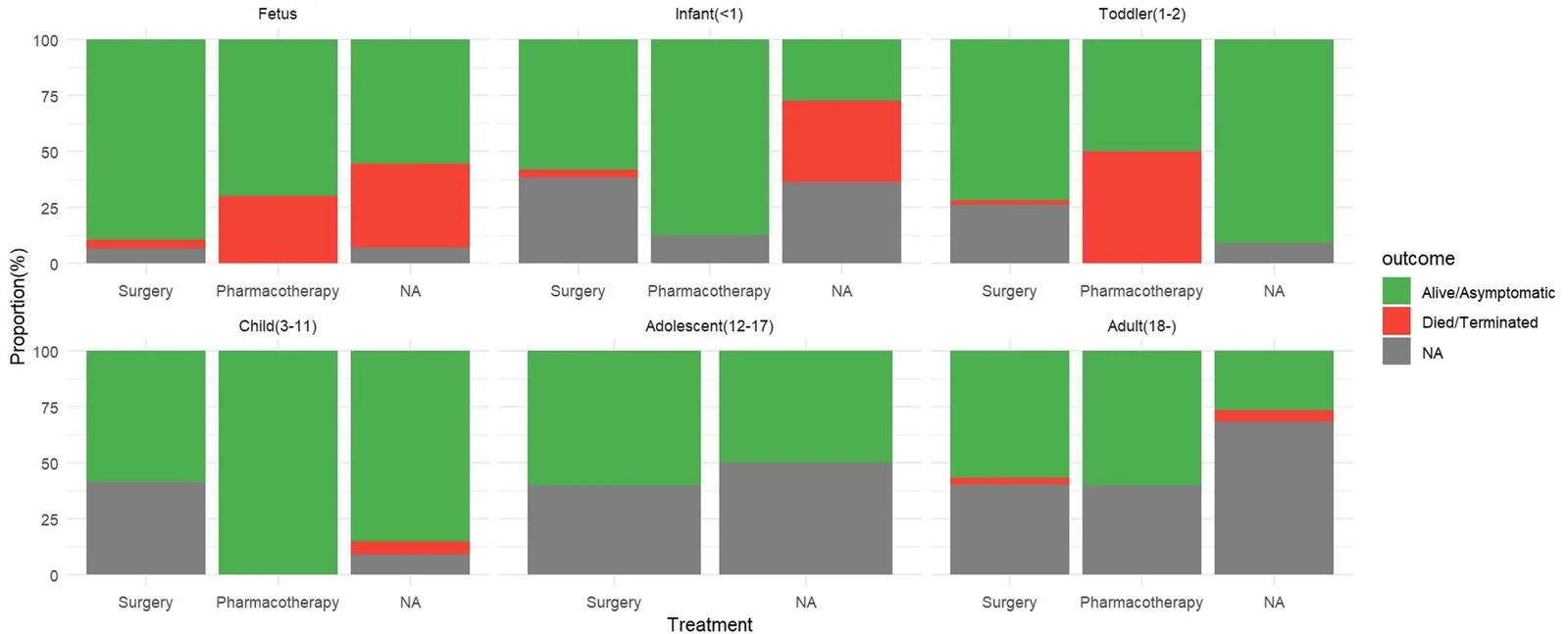
The association between treatment and outcome across age groups

Treatment strategy differed significantly by age (Table 1; Fig. 5). Surgical resection was performed in 44.6% of fetal cases (often post-termination or for severe symptoms), peaked in infancy (89.0%), and remained high in adolescents (84.9%) and adults (84.5%). Conservative management was uncommon across all groups (3.2% overall).

**Fig. 6:**
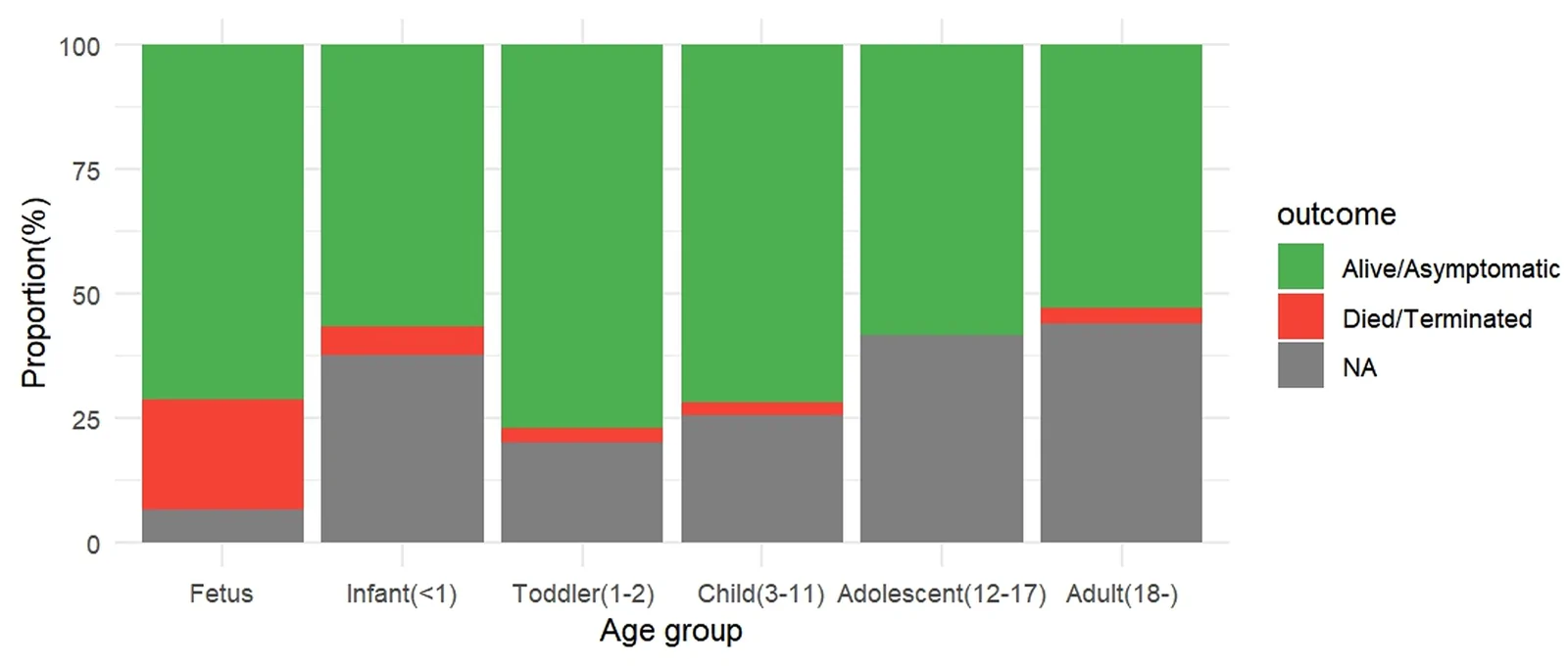
Distribution of outcomes by age group

Clinical outcomes also varied by age (Fig. 6). The composite rate of death, pregnancy termination, or disease recurrence was highest in the fetal group (22.1%; 95% CI 16.1%–29.2%), primarily driven by pregnancy terminations, and decreased sharply thereafter to 3.2% (95% CI 1.2%–6.9%) in adults (*p* < 0.001). Most postnatal patients were alive and asymptomatic at last follow-up.

Multivariable logistic regression identified clinical presentation at diagnosis as the only independent, statistically significant predictor of the composite adverse outcome (aOR = 10.73, 95% CI: 2.64–74.78, *p* = 0.004), after adjusting for age, sex, comorbidity, prenatal diagnosis, lesion location, gene mutation, and treatment (Table 4). This underscores that symptomatic disease at presentation, rather than age alone or the mere presence of a comorbidity, is a critical marker of higher risk.

**Table 4:** Adjusted odds ratios (aORs) for predictors of CPAM outcomes

Predictors	OR	95%CI	*P* value
Comorbidity	0.30	(0.06, 2.10)	0.180
Prenatal Diagnosed	3.86	(0.71, 21.13)	0.112
Age	0.87	(0.59, 1.25)	0.468
Sex	3.30	(0.98, 14.02)	0.073
Location	1.04	(0.38, 2.79)	0.942
Clinical Presentation	10.73	(2.64, 74.78)	0.004*
Gene Mutation	1.33	(0.18, 6.09)	0.739
Treatment	2.97	(0.48, 14.55)	0.201

*P value < 0.05

### Spectrum of genetic abnormalities and their association with clinical phenotypes

**Fig. 7:**
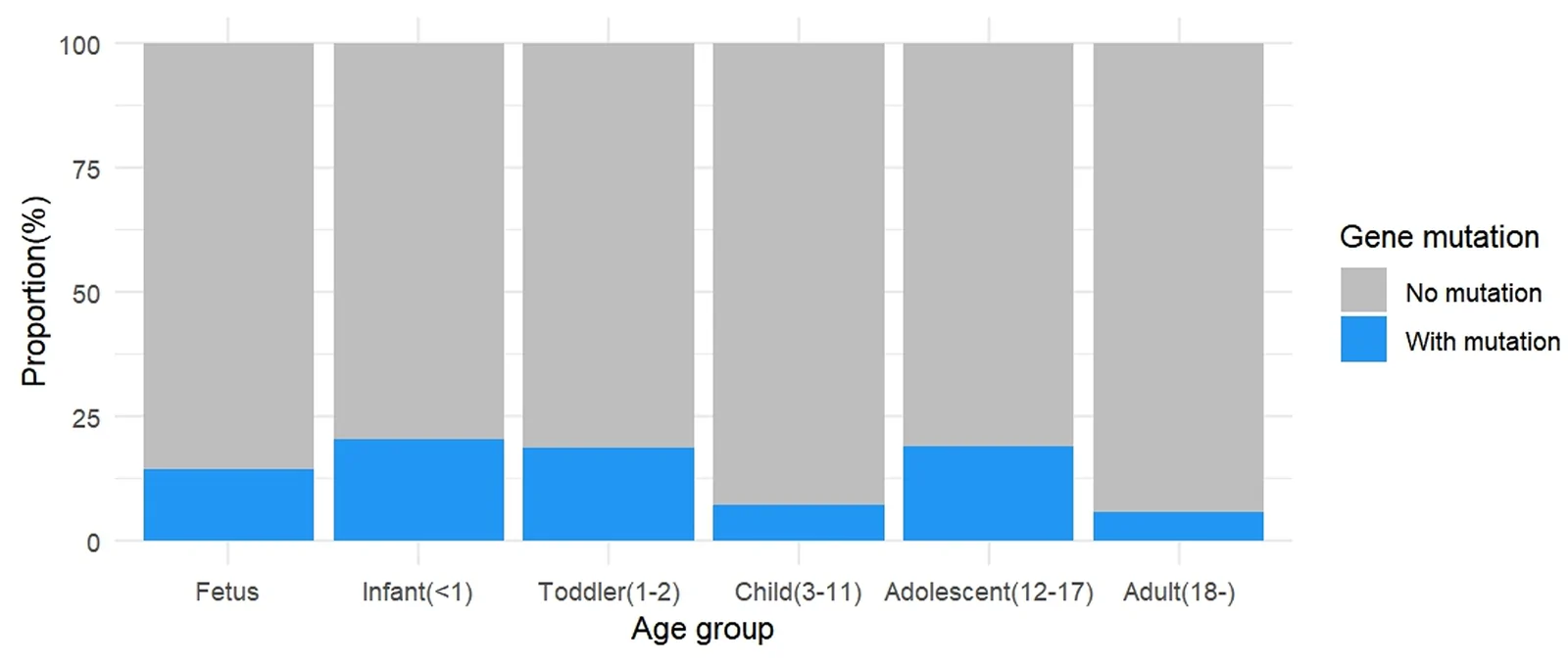
Distribution of gene mutations by age group

Within the overall cohort of 832 cases, genetic testing information was available for a subset of 119 individuals. Among these, detailed gene mutation status (e.g., specific variant classification) could be ascertained for 80 patients (14.3% of the total cohort). This mutation status did not show a significant association with clinical outcome (*p* = 0.739) or a clear differential distribution across age groups (Fig. 7).

A focused retrospective analysis was conducted on the 119 cases with any genetic test record. All samples were systematically categorized as genetically normal or abnormal based on the diagnostic significance of their reports (Table 5). Overall, 63.0% (95% CI: 53.7%–71.7%) of these tested individuals harbored genetic findings of potential clinical significance. Clinically, the vast majority of patients (33.6%, 95% CI: 25.8%-42.5%) had documented comorbidities, while 26.1% (95%CI: 19.0%-34.6%) had clinical presentation.

**Table 5:** Detailed classification of genetic test results in 119 cases

Category	Count *n*(%)	Specific Findings
**Negative/Normal Genetic Findings**	44(36.9)	Normal karyotype/WT
**Abnormal Genetic Findings**		
Gene Mutation/Fusion	45(37.8)	*KRAS G12D/V, TP53, FGFR2* mutation
Chromosomal Abnormality	9(7.6)	Trisomy 13, Klinefelter syndrome, Large CNVs
Gene Copy Number Variants	5(4.2)	Deletion/Duplication of *PMP22, HNF1B*
Other Gene Findings	16(13.5)	Vague entries (e.g., VUS, gene lists only)

The association between genetic status and basic clinical phenotypes was analyzed in the 119 tested cases. The genetically abnormal group had a higher proportion of individuals with comorbidities (50.0% vs. 8.5%) and symptomatic presentations (34.7% vs. 12.8%) compared to the genetically normal group.

## Discussion

This study of 832 CPAM cases, spanning from prenatal diagnosis to adulthood, provides a lifespan perspective on the clinical evolution of this rare congenital lung malformation. Our findings reveal CPAM not as a static fetal anomaly but as a dynamic condition whose comorbidity burden, symptomatic presentation, and associated risks undergo transformation across age groups. The results show an escalating risk of malignant transformation in adulthood, a distinct shift from incidental to symptomatic detection with age, and the identification of clinical presentation as a paramount independent prognostic factor, which may challenge the predominantly pediatric frame of reference that has long guided CPAM management. This study underscores the critical necessity of transitioning to a lifelong care model for individuals with CPAM.

### The escalating oncogenic risk: a call for adult surveillance protocols

The most striking and clinically significant finding of our study is the age-dependent increase in the prevalence of malignant and pre-malignant lesions. While present in only 2.9% of prenatally diagnosed cases, this risk rose to 7.7% in the pediatric population (0–18 years) and surged to 23.9% in adults. This progression confirms and quantifies the long-hypothesized oncogenic potential of CPAM (Fig. 3). The predominance of mucinous adenocarcinoma (MAC) across all age groups [22, 23, 30], including its presence even in infancy [31], aligns with the established pathogenetic link between CPAM (particularly Type 1) and this specific carcinoma, thought to arise from mucinous cell proliferation within the cysts [32]. Unlike MAC, bronchioloalveolar carcinoma (BAC) is mostly diagnosed after birth, further demonstrating the diversity of clinical manifestations of related malignant tumors [33–35].

This gradient of risk carries some implications. The traditional paradigm of CPAM management, largely focused on resolving neonatal respiratory symptoms or preventing childhood infections [12, 36–38], is insufficient for the adult patients. An asymptomatic adult with a known, unresected CPAM lesion or one diagnosed incidentally on imaging can no longer be considered at low risk [13, 33, 39]. The results suggest that the residual malformed lung tissue may serves as a predisposing field for carcinogenesis over decades. Therefore, the absence of symptoms in an adult is not a marker of safety but potentially a period of silent risk accumulation. We advocate for the formal establishment of evidence-based, long-term surveillance protocols for adults with CPAM, whether previously resected (to monitor for recurrence or metachronous lesions) or conservatively managed [40]. Such protocols should include periodic low-dose CT imaging, the modality of choice for detecting early neoplastic changes within cystic lung lesions, with frequency tailored to individual risk factors such as lesion type, size, and smoking history [39].

### The evolving clinical face of CPAM: from silent anomaly to symptomatic disease

Complementing the shifting comorbidity profile is the equally dynamic evolution of clinical presentation. Our data delineate a clear trajectory: CPAM is primarily an incidental finding in the earliest stages of life (80.9% prenatally, 67.5% in children) but transitions to a symptomatic condition in a substantial proportion of adults (only 48.4% asymptomatic). This decline in silent presentation is mirrored by the postnatal emergence of infection-related symptoms, cough, and hemoptysis, driven by environmental exposure and pulmonary maturation after birth [41, 42]. The higher prevalence of hemoptysis in adults (6.4%) compared to children (1.4%) is particularly noteworthy. While it can result from infection or inflammation, its increased frequency in the age group also carrying the highest malignancy risk necessitates a high index of suspicion for neovascularization or tumor erosion as an underlying cause [43, 44]. Similarly, chest pain, a purely postnatal complaint, may signal complications like pneumothorax, infection, or, in older patients, pleural involvement by malignancy [45].

Conversely, the profile of prenatal complications—fetal hydrops, hydrothorax, and maternal polyhydramnios—is a hallmark of the fetal pathophysiology, often related to mass effect, mediastinal shift, and potential vascular compromise [46, 47]. These severe presentations account for the high rate of pregnancy termination and perinatal mortality in the fetal group (22.1%). Their resolution or non-occurrence postnatally marks a distinct shift in the disease’s clinical impact. This dichotomy emphasizes that the “severity” of CPAM is not a fixed attribute but is context-dependent, defined by different mechanisms at different life stages.

### Presentation as the keystone prognostic indicator

A pivotal finding from our multivariate analysis is that the clinical presentation at diagnosis emerged as the strongest and only statistically significant independent predictor of adverse outcome (aOR = 10.73, 95%CI: 2.64–74.78, *p* = 0.004), overshadowing factors like the presence of a comorbidity, age at diagnosis, or even treatment modality in our model. This result carries major clinical and prognostic weight.

Thus, the disease manifestations of CPAM closely reflect its possible biological aggressiveness and pathological consequences. A lesion that remains quiescent and asymptomatic may have a more indolent natural history, regardless of its histological type or the patient’s age [48]. In contrast, a lesion that presents with respiratory distress, significant infection, or hemoptysis has already demonstrated its capacity to cause morbidity, likely reflecting larger size, strategic location, communication with the airway, or the presence of complicating factors like occult malignancy [49]. Therefore, the symptomatic patient, whether infant or adult, enters a higher-risk category that demands more urgent and definitive intervention. This insight can refine preoperative counseling and shared decision-making, move to a more nuanced strategy where the symptomatic status is a key determinant of management urgency.

However, this finding should be interpreted with caution against the backdrop of the same publication bias. Adults with asymptomatic or incidentally detected CPAM are massively underrepresented in the published literature, whereas those presenting with symptoms and subsequent adverse outcomes are preferentially reported. The strong association between symptomatic presentation and adverse outcome may therefore be inflated by selection bias, and its true magnitude in an unselected CPAM population remains to be determined. Until prospective, population‑based data become available, symptomatic presentation should be regarded as a clinically useful but potentially confounded risk indicator.

### High genetic abnormality burden amidst prevalent asymptomatic status: implications for risk stratification and proactive surveillance

By integrating genetic data with basic clinical phenotypes, this study shows a dilemma in modern clinical genomics: the complex relationship between the high detection rate of genetic variants and the relative paucity of overt clinical phenotypes. In this cohort, nearly 60% of individuals carried a genetic abnormality, yet 66.6% were asymptomatic (Supplementary Table 3). This observation necessitates a deeper reflection on the clinical significance of genetic findings, disease penetrance, and preventive medicine strategies. Firstly, the high proportion of “asymptomatic” individuals (66.6%) requires careful interpretation, which may encompass several scenarios: For identified definitive pathogenic mutations (e.g., *KRAS G12D/V*,* TP53* truncating mutations), the corresponding diseases (e.g., malignancies) have significant latency periods [50, 51]. Such results should be regarded as markers of high-risk status rather than a guarantee of current health, underscoring the potential value of early genetic screening in at-risk populations to initiate active surveillance [52–54]; the phenotypic expression of many genetic variants, particularly Variants of Uncertain Significance (VUS) in the “Other Genetic Findings” category and some low-penetrance alleles, is profoundly influenced by modifier genes, environmental factors, and epigenetics [55, 56]. Their sole presence is insufficient to cause recognizable symptoms, explaining the coexistence of numerous abnormal results with current asymptomatic status; “Asymptomatic” may only indicate a lack of significant complaints, while individuals may already have abnormal biochemical markers, subtle imaging findings, or other uncaptured subclinical phenotypes [57]. Second, the observed trend within the comorbidity subgroup—a higher proportion of individuals with genetic abnormalities (50.0% vs. 8.5%)—is highly suggestive. These comorbidities could be: for direct or indirect Consequences of Genetic Variants, chromosomal abnormalities or certain CNVs often lead to multisystem syndromes manifesting as complex comorbidities. Genetic background may increase an individual’s risk for common comorbidities like hypertension or diabetes, which manifest as disease when interacting with environmental factors. Meanwhile, some comorbidities may be unrelated to the core genetic diagnosis, reflecting the baseline disease burden in the population.

### Strengths and limitations

The principal strength of this study lies in its systematic integration and age-stratified pooled analysis of individual patient data from 832 CPAM cases, assembled through a pooled analysis of published literature, which provides a rare lifespan perspective on this condition. By aggregating individual patient data from numerous studies across several decades and geographic regions, we were able to analyze a sample size and age diversity typically unattainable in single-center studies [58, 59]. This pooled analysis design specifically enabled the capture of a wide clinical spectrum, including important but often underrepresented subgroups such as prenatally diagnosed cases, asymptomatic individuals, and adults, who are less frequently the focus of surgical series [8, 60, 61]. Our age-stratified, event-based analysis of comorbidities and presentations provides a granular, longitudinal view unavailable in smaller studies [62].

However, several important limitations must be acknowledged. First, the retrospective nature of the study and the heterogeneity of the aggregated data pose certain constraints. The data were originally collected by numerous teams over many years for clinical rather than research purposes, leading to inevitable variability in diagnostic criteria, imaging protocols, pathological review, and completeness of follow-up [63]. This variability, combined with the largely unavailable detailed data on crucial modifiers (e.g., cyst size, precise location, smoking history), limits the potential for more refined risk stratification and complicates the interpretation of treatment patterns and outcomes, which are subject to significant confounding. Second, as with many literature-based pooled analyses [64, 65], reliance on original reports without centralized re-review introduces potential inconsistencies in case classification and variable definitions (e.g., “asymptomatic” status, specific comorbidity types). Second, the analytical approach has inherent constraints. The classification of “comorbidity events” was necessary to capture case complexity but precludes precise patient-level risk calculation. Similarly, our outcome analysis is limited by variable and often short follow-up durations [66], particularly for adult cases sourced from the literature. Finally, while a pooled analysis mitigates some forms of bias by aggregating diverse sources, the present study remains subject to publication bias [67]. All frequencies and percentages reported herein represent the distribution documented in published cases only and may not be directly extrapolated to the general CPAM population. In reality, the majority of CPAMs are detected prenatally during routine second‑trimester ultrasound screening, and a substantial proportion (30–75%) undergo spontaneous regression or remain completely asymptomatic, thus seldom becoming the subject of a case report. Consequently, the true proportion of prenatal diagnoses in the general CPAM population is far higher than the 20.2% observed in our dataset, and the lifetime risks of malignancy and symptomatic progression are likely overestimated. The age‑stratified comorbidity and presentation profiles we describe therefore reflect the clinical spectrum of CPAM cases that came to medical attention and were considered sufficiently noteworthy for publication, not the natural history of all individuals living with CPAM. This fundamental ascertainment bias must be borne in mind when interpreting every quantitative estimate derived from this study. To overcome these limitations and precisely map the dynamic trajectory from genetic risk to clinical phenotype, future prospective cohort studies integrating multi-omics data and structured long-term follow-up are essential. A dedicated CPAM registry, collecting standardized longitudinal data from fetal life through adulthood, would be a critical step towards shifting from reactive treatment to proactive, lifelong health management.

### Clinical and public health implications

Despite these limitations, our findings have direct and actionable implications. Firstly, our findings offer tangible guidance for patients, families, and clinicians. Families can be informed that while fetal CPAM carries risks such as hydrops and associated anomalies [68, 69], the long-term outlook for children surviving the perinatal period is generally favorable, with a low immediate malignancy risk. However, the potential for later-life complications should be discussed as part of lifelong healthcare planning. For asymptomatic children, the decision for prophylactic resection remains nuanced [1, 70]; while the absolute malignancy risk in childhood is low (7.7%), it is not negligible and must be balanced against surgical risks, considering lesion characteristics (e.g., Type I cysts carry higher risk) and family preference. For adults, a critical implication is the need for heightened awareness among pulmonologists, thoracic surgeons, and primary care physicians that CPAM can be an antecedent to lung cancer [71]. Any adult with a history of CPAM (resected or not) presenting with new respiratory symptoms, especially hemoptysis, requires prompt and thorough investigation. We propose considering a baseline CT scan in early adulthood for all individuals with a history of CPAM to establish a reference point for future surveillance. This age-dependent shift in clinical threats—from mechanical complications in fetuses to infection in childhood and occult neoplasia in adults—reinforces the need for a dynamic, lifespan-oriented management approach.

Secondly, a possible implication arising from the limitations of our retrospective pooled analysis is the urgent need to establish national or international standardized prospective registries for CPAM just like Europe did [29]. Such registries would systematically collect longitudinal clinical, imaging, pathological, and genetic data from the fetal period through adulthood, thereby transforming fragmented past evidence into a driver for future precision medicine. Their core value is threefold: providing individuals with dynamic, personalized risk assessments and surveillance plans; generating high-level evidence to resolve clinical controversies (e.g., optimal timing of surgery for asymptomatic cases [70, 72]); and creating a high-quality biorepository to facilitate biomarker discovery for critical events like malignant transformation. Promoting the development of this public health data infrastructure is a crucial step towards shifting from reactive treatment to proactive, lifelong health management for individuals with CPAM.

Finally, our integrated analysis underscores that CPAM must be recognized as a chronic condition requiring a structured transition from pediatric to adult care services. Genetic findings should be leveraged as a key tool for risk stratification [73]. For asymptomatic individuals carrying high-penetrance pathogenic mutations, evidence-based long-term monitoring is warranted. For those with variants of uncertain significance (VUS), careful genetic counseling and periodic re-evaluation are recommended [74]. Future research must prioritize prospective, longitudinal cohorts with standardized imaging and biobanking to identify molecular biomarkers of malignant transformation and to finally resolve the debate on optimal timing of surgery through well-designed prospective studies.

## Conclusion

This study provides an empirical foundation for a fundamental paradigm shift in the understanding and management of CPAM—from a pediatric surgical anomaly to a lifelong chronic condition with age-specific risks. Our findings mandate the urgent development of comprehensive management frameworks designed to address the evolving risks and care needs across all life stages, which must include risk-adapted surveillance strategies for adults, symptom-driven clinical pathways, and integrated transition systems from pediatric to adult healthcare. To translate this paradigm into improved outcomes, future research must prioritize prospective cohorts with longitudinal biospecimen collection. Implementing this holistic framework is essential to optimize health outcomes for individuals with CPAM across their entire lifespan.

## Supplementary Information

Below is the link to the electronic supplementary material.

Supplementary Material 1

## Data Availability

Those datasets generated and/or analysed during the current study are not publicly available but are available from the corresponding author on reasonable request.

## Abbreviations

CPAM: Congenital Pulmonary Airway Malformation
CCAM: Congenital Cystic Adenomatoid Malformation
MAC: Mucinous Adenocarcinoma
BAC: Bronchioloalveolar Carcinoma
VUS: Variants of Uncertain Significance
aOR: Adjusted Odds Ratio
