# Supplementary material for: A lifespan pooled analysis of 832 cases: characterizing the lifespan profile of clinical presentations and comorbidities in congenital pulmonary airway malformation
Source: Orphanet J Rare Dis. 2026 Jul 1;21:327. doi: 10.1186/s13023-026-04464-6 (PMC13602632; doi:10.1186/s13023-026-04464-6)
Supplement: Supplementary file 1 [file 13023_2026_4464_MOESM1_ESM.docx]

**Supplementary Table legend**

Supplementary Table 1 Spectrum of Presentation with CPAM by prenatal/postnatal

Supplementary Table 2 Spectrum of Comorbidities with CPAM by prenatal/postnatal

Supplementary Table 3 Association Analysis Between Genetic Status and Clinical Phenotypes

**Supplementary Table 1 Spectrum of Presentation with CPAM by prenatal/postnatal**

| **Primary Presentation n (%) *** | **Total CPAM cases**  **(n = 832)** | **Prenatal CPAM**  **(n = 168)** | **Postnatal CPAM**  **(n = 664)** | | **P value** |
| --- | --- | --- | --- | --- | --- |
| **Asymptomatic / Incidental Finding** | **555(66.6)** | **136(80.9)** | **419(63.1)** | | **<0.001** |
| **Respiratory Distress (General)** | **54(6.5)** | **7(4.2)** | **47(7.1)** | **<0.001** | |
| **Infection-Related Presentation** | **59(7.1)** | **4(2.4)** | **55(8.3)** | **<0.001** | |
| - Pneumonia / recurrent infection | 54(6.5) | 1(0.6) | 53(8.0) |  | |
| - Fever | 5(0.6) | 3(1.8) | 2(0.3) |  | |
| **Cough** | **36(4.3)** | **0(0)** | **36(5.4)** | **<0.001** | |
| - Cough alone / recurrent cough | 21(2.5) | 0(0) | 21(3.2) |  | |
| - Cough combined with other symptoms | 15(1.8) | 0(0) | 15(2.3) |  | |
| **Hemoptysis** | **17(2.1)** | **0(0.0)** | **17(2.6)** | **<0.001** | |
| - Hemoptysis alone | 6(0.7) | 0(0) | 6(0.9) |  | |
| - Hemoptysis with other symptoms | 11(1.4) | 0(0) | 11(1.7) |  | |
| **Prenatal / Fetal & Maternal Complications** | **21(2.5)** | **19(11.3)** | **2(0.4)** | **<0.001** | |
| - Fetal hydrops / History of fetal hydrops | 8(0.9) | 7(4.2) | 1(0.2) |  | |
| - Polyhydramnios / Oligohydramnios | 4(0.5) | 4(2.4) | 0(0) |  | |
| - Major structural / chromosomal anomalies | 6(0.7) | 6(3.6) | 0(0) |  | |
| - Maternal complications | 3(0.4) | 2(1.2) | 1(0.2) |  | |
| **Chest Pain / Discomfort** | **13(1.5)** | **0(0)** | **13(2.0)** | **<0.001** | |
| **Pneumothorax** | **10(1.2)** | **2(1.2)** | **8(1.2)** | 0.093 | |
| **Other Single Presentations** | **67(8.0)** | **0(0.0)** | **67(10.1)** | **<0.001** | |

**Supplementary** **Table 2 Spectrum of Comorbidity events with CPAM by prenatal/postnatal**

| **Comorbidity Event n (%) *** | **Total CPAM**  **(n = 832)** | **Prenatal CPAM**  **(n = 168)** | **Postnatal CPAM**  **(n = 664)** | | **P value** |
| --- | --- | --- | --- | --- | --- |
| **No Comorbidity** | **521(62.6)** | **98(58.3)** | **423(63.7)** | | **<0.001** |
| **Malignant / Pre-malignant Lesions** | **81 (9.7)** | **5 (2.9)** | **76(11.4)** | **<0.001** | |
| - Mucinous adenocarcinoma (MAC) | 55 (6.6) | 5 (2.9) | 50(7.5) |  | |
| - Bronchioloalveolar carcinoma (BAC) | 26 (3.1) | 0 (0.0) | 26(3.9) |  | |
| **Pulmonary Sequestration** | **34 (4.1)** | **16 (9.5)** | **18(2.8)** | **0.027** | |
| - Extralobar pulmonary sequestration | 16 (1.9) | 10 (5.9) | 6(0.9) |  | |
| - Intralobar pulmonary sequestration | 18 (2.2) | 6 (3.6) | 12(1.9) |  | |
| **Cardiovascular Anomalies** | **19 (2.3)** | **2 (1.2)** | **17(2.6)** | 0.410 | |
| - Congenital heart disease / malformation | 11 (1.3) | 2 (1.2) | 9(1.4) |  | |
| - Other cardiovascular anomalies | 8 (1.0) | 0 (0.0) | 8(1.2) |  | |
| **Infections & Inflammatory Conditions** | **25 (3.0)** | **0 (0.0)** | **25(3.8)** | **<0.001** | |
| - Pneumonia / lung abscess / empyema | 10 (1.2) | 0 (0.0) | 10(1.5) |  | |
| - Tuberculosis / Nontuberculous mycobacteria | 5 (0.6) | 0 (0.0) | 5(0.8) |  | |
| - Aspergillosis / Aspergilloma | 5 (0.6) | 0 (0.0) | 5(0.8) |  | |
| - Others (CMV, fungus colonization, etc.) | 5 (0.6) | 0 (0.0) | 5(2.8) |  | |
| **Other Cysts / Structural Anomalies** | **14 (1.6)** | **4 (2.4)** | **10(1.5)** | 0.810 | |
| - Bronchogenic / esophageal | 7 (0.8) | 3 (1.8) | 4(0.6) |  | |
| - Arachnoid / other cysts | 7 (0.8) | 1 (0.6) | 6(0.9) |  | |
| **Pneumothorax** | **11 (1.3)** | **0 (0.0)** | **11(1.7)** | **0.003** | |
| - Spontaneous pneumothorax | 6 (0.7) | 0 (0.0) | 6(0.9) |  | |
| - Other / unspecified pneumothorax | 5 (0.6) | 0 (0.0) | 5(0.8) |  | |
| **Pleuropulmonary Blastoma (PPB)** | **5 (0.6)** | **0 (0.0)** | **5(0.8)** | **0.024** | |
| **Other Single Comorbidities** | **142 (17.1)** | **43 (25.6)** | **99(14.9)** | 0.780 | |

**Due to the presence of multiple comorbidities in some cases, we categorized and summarized the data based on the number of comorbid events rather than the number of patients.*

**Supplementary Table 3 Association Analysis Between Genetic Status and Clinical Phenotypes**

| **Clinical Phenotype** | **Total Cases**  **(n=119)** | **Genetically Normal Group (n=47)** | **Genetically Abnormal Group (n=72)** |
| --- | --- | --- | --- |
| **Patients with Comorbidity** | 40(33.6) | 4(8.5) | 36(50.0) |
| **Symptomatic Patients** | 31(26.0) | 6(12.8) | 25(34.7) |
